# Ketamine and Ro 25-6981 Reverse Behavioral Abnormalities in Rats Subjected to Dietary Zinc Restriction

**DOI:** 10.3390/ijms21134791

**Published:** 2020-07-06

**Authors:** Bartłomiej Pochwat, Helena Domin, Anna Rafało-Ulińska, Bernadeta Szewczyk, Gabriel Nowak

**Affiliations:** 1 Maj Institute of Pharmacology, Polish Academy of Sciences, Department of Neurobiology, Laboratory of Trace Elements Neurobiology, Smetna street 12, 31-343 Krakow, Poland; rafalo@if-pan.krakow.pl (A.R.-U.); szewczyk@if-pan.krakow.pl (B.S.); 2 Maj Institute of Pharmacology, Polish Academy of Sciences, Department of Neurobiology, Smetna street 12, 31-343 Krakow, Poland; domin@if-pan.krakow.pl; 3 Department of Pharmacobiology, Faculty of Pharmacy, Jagiellonian University Medical College, 9 Medyczna Street, 30-688 Kraków, Poland

**Keywords:** ketamine, Ro 25-6981, zinc deficiency, depression

## Abstract

Clinical and preclinical studies indicate that zinc (Zn) is an essential factor in the development and treatment of major depressive disorder (MDD). Conventional monoamine-based antidepressants mobilize zinc in the blood and brain of depressed patients as well as rodents. N-methyl-D-aspartate acid receptor (NMDAR) antagonists exhibit antidepressant-like activity. However, not much is known about the antidepressant efficacy of NMDAR antagonists in zinc-deficient (ZnD) animals. We evaluated the antidepressant-like activity of two NMDAR antagonists (ketamine; global NMDAR antagonist and Ro 25-6981 (Ro); selective antagonist of the GluN2B NMDAR subunit) in ZnD rats using the forced swim test (FST) and sucrose intake test (SIT). A single dose of either Ro 25-6981 or ketamine normalized depressive-like behaviors in ZnD rats; however, Ro was effective in both tests, while ketamine was only effective in the FST. Additionally, we investigated the mechanism of antidepressant action of Ro at the molecular (analysis of protein expression by Western blotting) and anatomical (density of dendritic spines by Golgi Cox-staining) levels. ZnD rats exhibited decreased phosphorylation of the p70S6K protein, and enhanced density of dendritic spines in the prefrontal cortex (PFC) compared to control rats. The antidepressant-like activity of Ro was associated with the increased phosphorylation of p70S6K and ERK in the PFC. In summary, single doses of the NMDAR antagonists ketamine and Ro exhibited antidepressant-like activity in the ZnD animal model of depression. Animals were only deprived of Zn for 4 weeks and the biochemical effects of Zn deprivation and Ro were investigated in the PFC and hippocampus. The shorter duration of dietary Zn restriction may be a limitation of the study. However, future studies with longer durations of dietary Zn restriction, as well as the investigation of multiple brain structures, are encouraged as a supplement to this study.

## 1. Introduction

Major depressive disorder (MDD) is a serious medical problem that generates enormous economic and social costs globally. The morbidity associated with this disease has been on the increase [1,2,3] and therefore presents a considerable challenge in terms of effective treatment strategies.

There are still gaps in the understanding of the biological mechanisms underlying MDD, with no definitive therapy at the moment. Currently used monoamine-based antidepressant drugs are not effective in a high percentage of people who suffer from MDD [4]. Meanwhile, the crucial biological factors involved in the pathophysiology of MDD are not known. Therefore, new studies on more effective therapies for MDD and a better explanation of the social and biological causes of this illness are warranted.

Some evidence indicates that lifestyle, including diet, maybe a risk factor for the development of MDD [5]. Among others, decreased dietary intake of Zn may be a risk factor for the development of MDD. A few epidemiological reports have suggested an association between MDD and decreased dietary Zn intake [6,7]. Other studies have shown negative correlations between reduced serum Zn levels and the severity of MDD symptoms [8,9]. Additionally, decreased serum Zn levels have been reported in MDD patients [10,11,12,13]. Interestingly, in some MDD patients, blood Zn levels have been normalized after antidepressant treatment [14]. Both the lack of zinc normalization and reduced blood zinc levels have also been described in patients with refractory depression [9,15,16]. The role of Zn in the development of MDD symptoms is equally evident in preclinical paradigms. Chronic dietary Zn restriction evoked depressive-like symptoms in mice, with increased immobility time in the forced swim test (FST) and the tail suspension test [17,18,19].

Similar behavioral consequences of zinc deficiency have been reported in rats. Rats fed with a low Zn diet exhibited longer immobility time in the FST, reduced sucrose intake, and reduced social interactions [20,21,22]. Increased immobility time in the FST in ZnD rats was normalized by two weeks of treatment with the serotonin selective reuptake inhibitor (SSRI) fluoxetine [21]. However, it should be emphasized that the relationship between ZnD and the efficacy of particular antidepressant strategies is not fully understood.

Recently, ketamine and other NMDAR antagonists have been extensively evaluated as potential antidepressant drugs [23,24]. In clinical studies, a single dose of ketamine induced rapid and long-lasting antidepressant response [25,26]. What is very important and unique is that ketamine or its enantiomer *(S)*-ketamine was also effective in the treatment of resistant depression [25,26,27,28] and MDD with suicidal ideations [29,30,31]. Moreover, ketamine and other NMDAR antagonists such as Ro and MK-801 reversed behavioral abnormalities in animal models of depression [32,33]. Because ZnD causes behavioral abnormalities akin to chronic stress, we asked the question—do atypical glutamate-antidepressant compounds such as ketamine and Ro exhibit antidepressant-like activity in ZnD rats? This question is important because Zn is an endogenous antagonist of NMDAR and is released alongside glutamate from zincergic neurons in brain regions such as the hippocampus (Hp) and PFC, areas involved in mood regulation [34,35,36,37]. Furthermore, in contrast to classic antidepressants, NMDAR antagonists (ketamine, MK-801) induce an antidepressant-like response in GPR39 receptor knockout mice. GPR39 is an endogenous receptor that is positively modulated by Zn [38]. Therefore, these results suggest that NMDAR antagonists can also be active under conditions where zinc-dependent signaling pathways are disrupted. In other words, because NMDAR antagonists are effective in GPR39 knockout mice, they may likely function independently of Zn levels as well. To address the question concerning NMDAR antagonists in ZnD rats, we performed a few behavioral tests such as SIT, FST, and locomotor activity (LA) test following the administration of a single dose of either ketamine or Ro to rats exposed to 4 weeks of dietary Zn restriction. Based on our results, we chose Ro to carry out biochemical and anatomical studies. The antidepressant-like activity of Ro is dependent on the activation of intracellular signaling pathways such as extracellular regulated kinase (ERK), p70S6K-dependent synthesis of synaptic proteins, including synapsin I and the GluA1 subunit of glutamate α-amino-3-hydroxy-5-methyl-4-isoxazolepropionic acid receptor (AMPAR) acid [33,39,40]. Both the ERK and p70S6K signaling pathways are involved in the synthesis of synaptic proteins and remodeling of dendritic spines. Therefore, the goal of this study was to ascertain whether the same molecular mechanisms are responsible for the antidepressant-like activity of Ro in ZnD rats. Consequently, we measured the expression of these proteins in the PFC, Hp, and investigated the density of dendritic spines in the cingulate (Cg3) and infralimbic (IL) cortex.

## 2. Results

### 2.1. Effects of Ro and Ketamine on Immobility Time and Locomotor Activity of ZnD Rats

**Figure 1:**
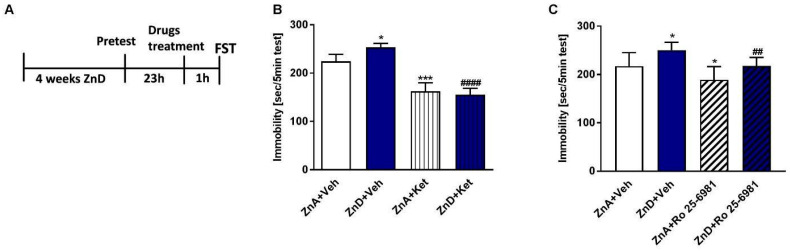
The effect of a single dose of ketamine (Ket) and Ro 25-6981 in the forced swim test (FST) in rats subjected to zinc deficiency. (**A**) Experimental schedule of drug treatments and behavioral test. Ketamine (10 mg/kg; *i.p.*), n = 8–10 (**B**) and Ro 25-6981 (10 mg/kg; *i.p.*), *n* = 8–9 (**C**) were administered 60 min before the FST. All data were analyzed by two-way ANOVA and Newman–Keuls multiple comparisons test. Values are expressed as mean ± S.E.M. Two way ANOVA for ketamine showed non-significant interaction [F (1, 32) = 1.794, *p* = 0.1899], the significant effect of ZnD [F (1, 32) = 35.4, *p* < 0.0001], and the non-significant effect of ketamine [F (1, 32) = 0.6554, *p* = 0.4242]. Two way ANOVA for Ro 25-6981 showed non-significant interaction [F (1, 31) = 0.1005, *p* = 0.7534], the significant effect of ZnD [F (1, 31) = 15.38, *p* = 0.0005], and the significant effect of Ro 25–6981 [F (1, 31) = 15.94, *p* = 0.0004]. * *p* < 0.05 vs. ZnA + Veh; *** *p* < 0.001 vs ZnA + Veh; ^####^ *p* < 0.0001 vs. ZnD + Veh; ^##^ *p* < 0.01 vs. ZnD + Veh. ZnA—zinc adequate diet; ZnD—zinc deficient diet.

ZnD rats exhibited increased immobility time in the FST; however, ketamine and Ro decreased the immobility time in these rats (Figure 1A–C). Both ketamine and Ro also reduced the immobility time in zinc-adequate (ZnA) rats compared to control rats. The effects induced by Ro in the ZnD rats could be nonspecific because of the increasing trend in locomotor activity observed after Ro administration (Table 1). However, ketamine did not exhibit a similar effect in the LA test (Table 1). Both atypical compounds (Ro and ketamine) showed potential antidepressant-like activity after a single dose.

Locomotor activity (distance traveled in cm) was recorded for 10 min. and presented as % of ZnA + NaCl. All data were analyzed by two way ANOVA and Newman–Keuls multiple comparisons test. Values are expressed as mean ± S.E.M, (*n* = 5–7). Two way ANOVA showed non-significant interaction [F (2, 30) = 0.2244, *p* = 0.8003], the significant effect of ZnD [F (2, 30) = 7.383, *p* = 0.0025], and the non-significant effect of treatment [F (1, 30) = 1.742, *p* = 0.1969]. Values are expressed as mean ± SEM (*n* = 5–7).

### 2.2. Effects of Ro and Ketamine on Sucrose Intake in Rats Subjected to Dietary Zn Restriction

**Figure 2:**
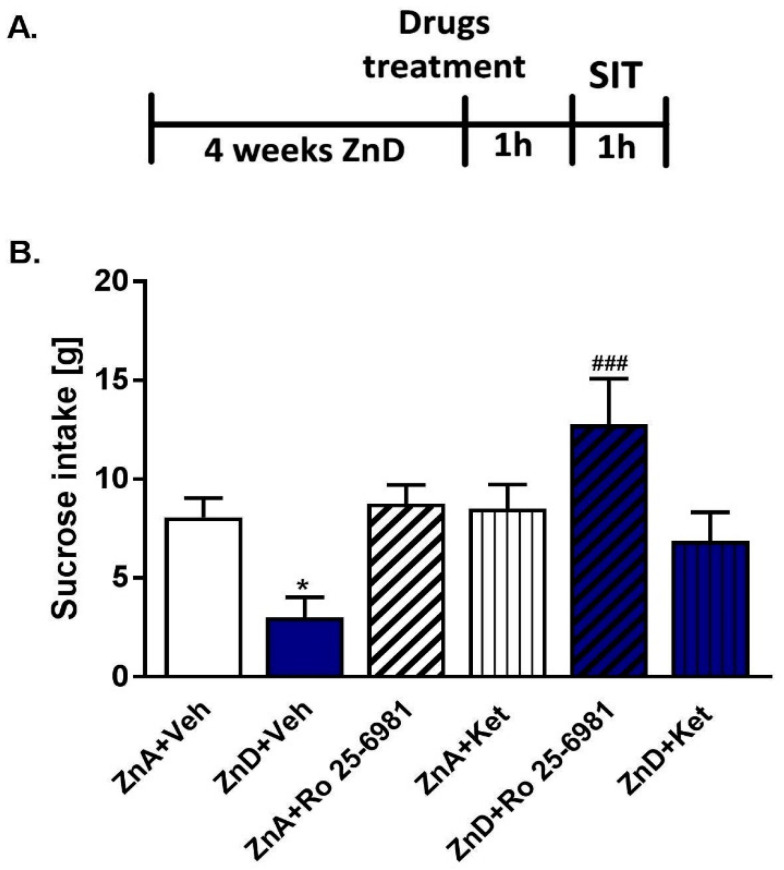
The effect of a single dose of ketamine (Ket) and Ro 25–6981 on sucrose intake in rats subjected to zinc deficiency. (**A**) Experimental schedule of drug treatments and behavioral test; (**B**) ketamine (10 mg/kg; *i.p.*) and Ro 25–6981 (10 mg/kg; *i.p.*) were administered 60 min before the test. Data were analyzed by two-way ANOVA and the Newman–Keuls multiple comparisons test. Values are expressed as mean ± S.E.M. Two-way ANOVA showed significant interaction [F (2, 44) = 5.555; *p* = 0.0071], the significant effect of ZnD [F (2, 44) = 7.268; *p* = 0.0019] and the non-significant effect of treatment [F (1, 44) = 0.6351, *p* = 0.4298]. * *p* < 0.05 vs. ZnA + Veh; ^###^ *p* < 0.001 vs. ZnD + Veh (*n* = 8–9).

To further evaluate the antidepressant potency of single doses of Ro and ketamine, we performed the SIT. Only Ro significantly reversed the reduced sucrose intake in ZnD rats (Figure 2A,B). Although ketamine followed a similar trend, the effect was not statistically significant. Based on these results, we chose to perform further biochemical assays with Ro.

### 2.3. Effects of Ro and ZnD on the Levels of Synaptic Proteins and ERK/p-70S6K Kinases

**Figure 3:**
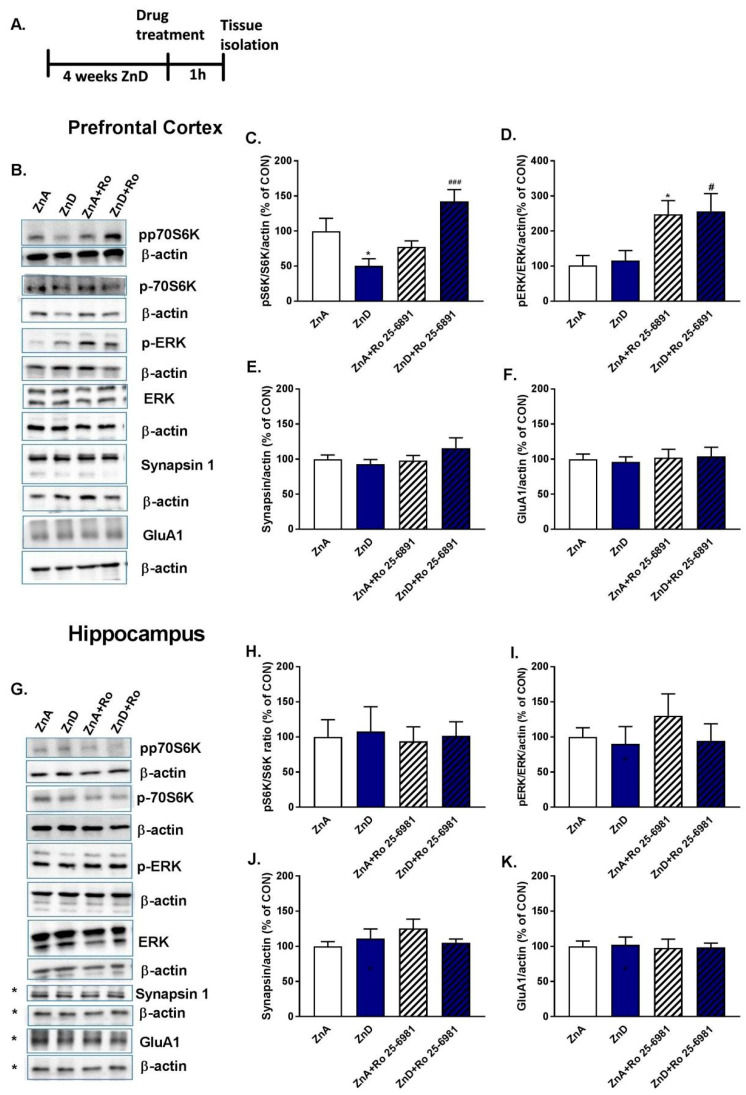
The effect of the administration of single dose of Ro 25–6981 on the expression of pS6K/S6K, p-ERK/ERK, synapsin I and GluA1 subunit of AMPA receptors in the prefrontal cortex (PFC) and hippocampus (Hp) of rats subjected to ZnD, *n* = 8–10; (**A**) experimental schedule; (**B**) representative blots for PFC; (**C**–**F**) protein expression in PFC; (**G**) representative blots for Hp, * images from the same part of the membrane; (**H**–**K**) protein expression in Hp. All data were analyzed by two-way ANOVA and the Newman–Keuls multiple comparisons test. All values are expressed as mean ±S.E.M. PFC: (**B**) pS6K/S6K: interaction [F(1,31) = 17.18, *p* = 0.0002]; effect of Ro 25–6981 [F (1, 31) = 6.335, *p* = 0.0172]; effect of ZnD [F (1, 31) = 0.3069, *p* = 0.5836]; * *p* < 0.05 vs. ZnA, ^###^ *p* < 0.001; (**C**) p-ERK/ERK: interaction [(1, 32) = 0.0063, *p* = 0.9372]; effect of Ro 25–6981 [F (1, 32) = 14.76, *p* = 0.0005]; effect of ZnD [F (1, 32) = 0.09003, *p* = 0.7661]; * *p* < 0.05 vs. ZnA, ^#^ *p* < 0.05 vs. ZnD; (**D**) Synapsin I: interaction [F (1, 31) = 1.981, *p* = 0.1692]; effect of Ro 25–6981: [F (1, 31) = 1.411, *p* = 0.2439], effect of ZnD [F (1, 31) = 0.3487, *p* = 0.5591]; **(E)** GluA1: Interaction [F (1, 36) = 0.07359, *p* = 0.7877]; effect of Ro 25–6981 [F (1, 36) = 0.2268, *p* = 0.6368], effect of ZnD [F (1, 36) = 0.01161, *p* = 0.9148]; **Hp: (H)** pS6K/S6K: interaction [F (1, 32) = 1.979 × 10^−6^, *p* = 0.9989]; effect of Ro 25–6981 [F (1, 32) = 0.05986, *p* = 0.8083]; effect of ZnD [F (1, 32) = 0.0851, *p* = 0.7724]; **(I)** p-ERK/ERK: interaction [F (1, 32) = 0.2892, *p* = 0.5944]; effect of Ro 25–6981 [F (1, 32) = 0.5073, *p* = 0.4815]; effect of ZnD [F (1, 32) = 0.8807, *p* = 0.3550]; **(J)** Synapsin I: interaction [F (1, 32) = 2.334, *p* = 0.1364]; effect of Ro 25–6981: [F (1, 32) = 0.899, *p* = 0.3502], effect of ZnD [F (1, 32) = 0.2022, *p* = 0.6560]; **(K)** GluA1: Interaction [F (1, 32) = 0.003627, *p* = 0.9523]; effect of Ro 25–6981 [F (1, 32) = 0.09773, *p* = 0.7566], effect of ZnD [F (1, 32) = 0.02311, *p* = 0.8801].

The antidepressant-like activity of Ro is dependent on the activation (by phosphorylation) of intracellular proteins such as ERK and p70S6K, the effectors of mTOR and MAPK (mitogen activated protein kinase) intracellular signaling pathways [39,40]. Moreover, p70S6K is involved in the process of neuroplasticity which is associated with changes in synaptic proteins such as synapsin I or the GluA1 subunit of AMPA receptors [39,40]. We determined the effects of Ro on the expression of these proteins in the PFC and Hp, brain structures crucial in mood regulation (Figure 3A,B,G). Figure 3C shows that dietary Zn restriction reduced the ratio of phospho-p70S6K (ph-p70S6K)/p70S6K in the PFC. Administration of Ro reversed these effects. In contrast, ph-p70S6K/p70S6K and phospho-ERK (p-ERK)/ERK ratios were not significantly altered by dietary Zn restriction (Figure 3D). However, Ro enhanced these ratios in the PFC of ZnA and ZnD rats (Figure 3D). No significant changes were seen in the expression of the rest of the proteins (Figure 3E–K).

### 2.4. Effects of Ro and ZnD on the Density of Dendritic Spines in the Medial Prefrontal Cortex (mPFC)

**Figure 4:**
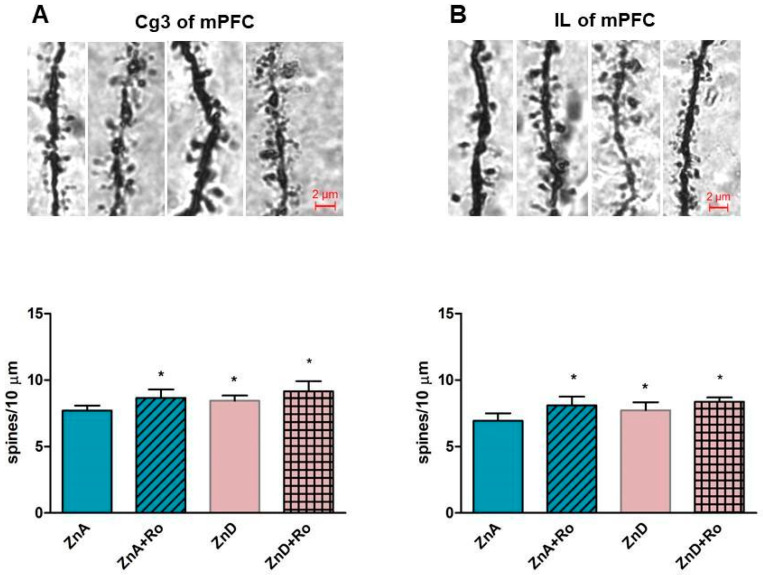
The effect of a single dose of Ro 25-6981 on density of dendritic spines in the Cg3 cortex (**A**) and IL cortex (**B**). Ro 25-6981 (10 mg/kg; *i.p.*) was administered 180 min before the decapitation, *n* = 5–6. Data were analyzed by two-way ANOVA and the Newman–Keuls multiple comparisons test. Values are expressed as mean ± S.E.M.; (**A**) two way ANOVA for Cg3 showed non-significant interaction [F (1, 17) = 0.2588, *p* = 0.6175], the significant effect of ZnD [F (1, 17) = 11.29, *p* = 0.0037], and the significant effect of Ro 25–6981 [F (1, 17) = 6.498, *p* = 0.0207]; (**B**) Two way ANOVA for IL showed non-significant interaction [F (1, 16) = 1.178, *p* = 0.2938, the significant effect of ZnD [F (1, 16) = 13.07, *p* = 0.0023] and the significant effect of Ro 25–6981 [F (1, 16) = 4.692, *p* = 0.0457]. *p < 0.05 vs. ZnA + Veh.

As shown in Figure 4A-B, ZnD enhanced the density of spines on the secondary and tertiary apical dendrites compared to ZnA rats in the Cg3 cortex (Figure 4A) and the IL cortex (Figure 4B). Administration of a single dose of Ro increased the density of dendritic spines in the Cg3 cortex (Figure 4A) and IL cortex (Figure 4B) of ZnA rats. We did not find statistically significant effects of Ro on dendritic spine density in the ZnD group, but there was a clear upward trend in ZnD rats treated with Ro.

## 3. Discussion

Both clinical and preclinical evidence indicates that lower intake of dietary of zinc may be associated with MDD symptoms in humans and depressive-like behaviors in rodents [9,15,19,21]. Also, the negative relationship between serum Zn levels and the response to monoamine-dependent antidepressant drugs in humans has been reported [9,15,16,21]. In contrast, a few preclinical studies have shown that chronic treatment with classic drugs such as fluoxetine or desipramine reversed the depression phenotype induced by dietary Zn restriction in rats and mice [19,21].

From a mechanistic standpoint, we have previously shown that dietary Zn restriction in rats reduced the levels of this microelement in the serum, Hp, and PFC of rats. The reduction was accompanied by increased glutamate release in the PFC after KCl stimulation [19,20,21,41]. Since Zn blocks NMDAR and is crucial for the proper functioning of glutamatergic/zincergic neurons in the hippocampus and PFC [34,35,36,37,42], we wanted to find out whether Zn depletion has an impact on the antidepressant response of glutamate-based atypical compounds. Thus, we examined the antidepressant-like effects of single doses of fast-acting, atypical compounds (Ro and ketamine) in rats subjected to 4 weeks of dietary Zn restriction. Both Ro and ketamine exert their primary effects through interaction with the glutamate system, albeit differently. Ro selectively blocks NMDAR containing the GluN2B subunit, while ketamine globally blocks NMDAR [43].

Here, we show that a single dose of ketamine and Ro reduced immobility time in ZnD rats in the FST. Because Ro, in contrast to ketamine, showed the trend to enhance locomotor activity, its antidepressant efficacy in the FST should be interpreted with caution, as these effects could be nonspecific. Because we previously found that ZnD rats exhibited reduced sucrose intake compared to ZnA animals [20], we further evaluated the antidepressant-like activity of both compounds in this behavioral test. We confirmed our earlier observations that dietary Zn restriction decreases sucrose intake. Moreover, we found that only Ro reversed the effect induced by ZnD in a statistically significant manner. While ketamine also increased sucrose intake in ZnD rats, the effect was not statistically significant. It is known that a single dose of either ketamine or other NMDAR antagonists like Ro induces antidepressant-like potency in preclinical stress-related paradigms [32,44,45]. On the other hand, classic antidepressant drugs like fluoxetine or desipramine are effective only after chronic treatment [19,21]. Thus, our results suggest that ZnD may serve as a potential animal model of depression with similar predictive validity to the better-known stress-dependent paradigms.

We also wanted to find out what kinds of mechanisms underlie the antidepressant-like activity of NMDAR in ZnD rats. Because Ro was more potent than ketamine in the behavioral tests, we used Ro for our biochemical experiments. There are indications that the potential antidepressant mechanism of Ro involves the activation of intracellular signaling pathways such as ERK and mTOR/p70S6K and the enhanced expression of synaptic proteins, including synapsin I or the GluA1 subunit of AMPAR [32,39]. The expression and activation of these proteins are crucial for the processes of neuroplasticity, which are perturbed in animal models of depression [23]. Moreover, the disruption of neuroplasticity defined as a decrease in synaptic plasticity-related proteins and reduced density of synaptic spines has been reported in ZnD mice, induced by the chronic administration of clioquinol, a known Zn/copper chelator [46]. Since a dysfunction in neural plasticity has been reported in the Hp and PFC in animal models of depression [47,48], we chose these same regions for our assays. Our results reveal that ZnD did not alter the expression of the GluA1 subunit of AMPAR, synapsin I, and p-ERK/ERK ratio in the Hp or PFC. However, the ratio of ph-p70S6K/total p70S6K was significantly reduced in the PFC but not Hp of ZnD rats. Importantly, a single dose of Ro restored the ratio of ph-p70S6K/total p70S6K in ZnD rats. p70S6K regulates the translation of synaptic proteins like the GluA1 subunit of AMPAR or synapsin I. Our results do not reveal any effect of Ro on the expression of synaptic proteins.

Nevertheless, we should mention that the brains used for protein expression studies were harvested 1h after Ro treatment. Ro’s influence on the expression of synapsin I and the GluA1 subunit are seen after 6 h after its administration [39]. Therefore, the effects induced by Ro may occur later. Because the reduced activity of p70S6K may be associated with the decreased density of dendritic spines, and that NMDAR antagonists increase the density of dendritic spines in animal models of depression and naive rodents [49,50,51], we subsequently investigated the effects of ZnD and Ro on this parameter in the medial prefrontal cortex (mPFC). Contrary to some other preclinical depression paradigms [49,50,51], ZnD increased spine density in the mPFC (IL and Cg3 cortex). In addition, Ro elevated spine density in control animals in a statistically significant manner. This trend was also observed in ZnD rats following treatment with Ro; however, this was not statistically significant. These outcomes may suggest that the reduction in dendritic spine density in the mPFC is not involved in the induction of depressive-like behaviors observed in ZnD rats.

Additionally, our studies showed that zinc deficiency induced by dietary Zn restriction evoked different brain changes compared to that caused by chelators in mice (decreased density of spines). Another aspect of the studies was the lack of association between spine density and the reduced ratio of ph-p70S6K/totalp70S6K in ZnD rats. This phenomenon can be explained by the fact that spine density can be controlled by the interplay between many intracellular signaling pathways, such as the ERK mTOR/p70S6K pathway [52]. Therefore, the activity of ERK kinase is also very important for dendritic spine formation. In ZnD rats, we did not observe alterations in the ratio of phospho-ERK/total ERK. In stress-related paradigms, the reduction in cortical spine density correlated with decreased levels of ERK and p70SK phosphorylation [52,53,54]. Thus, it is very plausible that proper dendritic spine density requires the interaction of ERK and S6K kinases. Recently, we showed that ERK phosphorylation is indispensable for Ro antidepressant-like activity [40]. Our results show that Ro treatment results in an enhanced ERK phosphorylation in both ZnD and ZnA rats.

In summary, we have confirmed our previous observations that ZnD induced by dietary Zn restriction evokes depressive-like behaviors in rats. These behavioral abnormalities are reversed by the administration of a single dose of the NMDAR antagonist ketamine or Ro. The antidepressant-like activity of Ro was associated with the activation of p70S6K and ERK kinases. Our results also confirm that NMDAR antagonists activate intracellular signaling pathways involved in the processes of neuroplasticity. However, the precise role of this signaling pathway in the formation of dendritic spines in ZnD rats requires further studies. The current studies are important from a clinical standpoint, as they focus on the fact that diet can be an important risk factor in the development of psychiatric disorders like MDD. Moreover, these studies again confirmed that a single dose of an NMDAR antagonist elicits antidepressant efficacy in preclinical conditions.

## 4. Material and Methods

### 4.1. Animals

The experiments were carried out on male Sprague-Dawley rats (Charles, River, Germany) kept under standard laboratory conditions of lighting (light phase: 7:00–19:00) and temperature (19–21 °C), with free access to water and food. All manipulations were carried out between 8:00 and 16:00. All procedures in this study were performed according to the guidelines of the European Community Council (Directive 86/609/EEC). The study was approved by the Ethics Committee of the Maj Institute of Pharmacology at the Polish Academy of Sciences in Krakow (87/2016, 31.05.2016). All efforts were made to minimize animal suffering and to reduce the number of animals used.

### 4.2. Drug Administration

The following drugs were used: ketamine (10 mg/kg) (Biowet Puławy, Poland) was administered intraperitoneally (*i.p*); Ro 25-6981 maleate (10 mg/kg) (Tocris, Bristol, UK) dissolved in 10% DMSO (Sigma Aldrich, St.Louis, MO, USA) was administered *i.p*; the control groups received physiological saline (0,9% NaCl) (*i.p*) or 10% DMSO *(i.p)*. For behavioral experiments, drugs were administered one hour before each test. For Western blotting assays, drugs were also administered 1 h before decapitation. Dendritic spine analysis was carried out in rats subjected to drug treatment 3 h before decapitation. Drug doses were selected based on our preliminary studies and published data [39,40].

### 4.3. Forced Swim Test (FST)

The FST was carried out as previously described [55]. On day one of the experiment, animals were individually placed in plexiglass cylinders (40 cm in height, 18 cm in diameter), containing 25 cm of water maintained at 24–25 °C for a 15 min habituation period. After removal from water, animals were returned to their home cages. On the second day, the rats were placed again in the cylinders, and the total duration of immobility was measured for a 5 min test period.

### 4.4. Locomotor Activity

Because some compounds may give non-specific responses in the FST (decreased immobility time evoked by enhanced general locomotor activity), spontaneous locomotor activity was recorded individually for each animal in Opto-Varimex cages (Columbus Instruments, USA) linked online to a compatible IBM PC. Each cage (43 × 44 × 25 cm) was surrounded with a 15 × 15 array of photocell beams located 3 cm from the floor surface. Interruptions of these photo beams resulted in horizontal activity defined as the distance traveled. Locomotor activity was recorded for 10 min and analyzed using the Auto-track software (Columbus Instruments, USA) and presented as the distance traveled in cm.

### 4.5. Zinc Restriction

During the habituation phase, rats were fed a standard diet with 35 mg Zn/kg. Following the habituation phase, the animals were divided into two groups; each group was fed a zinc adequate diet of 50 mg Zn/kg or a zinc-deficient diet of 3 mg Zn/kg for 4 weeks. The animals were further used in behavioral tests (experimentally naive subjects were used only once in each behavioral test) or biochemical analysis. Diets were purchased from Altromin GmbH (Lage, Germany). The animals were housed 5 per cage (except for the rats used in the sucrose intake test, which were housed individually) in a controlled environment (temperature 22 ± 2 °C, 12 h light/dark cycle, 40–50% humidity) with free access to food and water.

### 4.6. Sucrose Intake Test

Individually housed rats were fed the ZnA or ZnD diet for 4 weeks, following which the SIT was carried out. A test session was preceded by a training session. Both the training and the test sessions were conducted in home cages that were equipped with two bottles; one bottle contained a 1% sucrose solution, and the other contained sterile water. In the training session, the animals were trained to consume the 1% sucrose solution for 48 h. To prevent the possible effects of side preference, the positions of the bottles were switched every 12 h. Following the training session, sterile water was provided for 6 h, while withholding food and water for the next 18 h. The test session was performed 24 h after the training session and lasted 1 h. Rats were given a choice between sterile water and the 1% sucrose solution. The sucrose intake of the rats was measured by weighing the pre-weighed bottles at the end of the test.

### 4.7. Synaptosome Preparation and Western Blotting

Tissue samples were dissected from the PFC and homogenized in ice-cold lysis buffer (A) [0.32 M sucrose, 20 mM HEPES (pH 7.4) (Bioshop, Burlington, OA, Canada); 1 mM EDTA (Sigma Aldrich, St. Louis MO, USA); 1X protease inhibitor cocktail (Sigma Aldrich, St. Louis, MO, USA), 5 mM NaF (Sigma Aldrich, St. Louis, MO, USA) and 1 mM NaVO3 (Sigma Aldrich, St. Louis, MO, USA)]. Homogenates were then centrifuged at 2800 rpm for 10 min at 4 °C. The resultant pellet (nuclear fraction) was resuspended in buffer A, and the supernatant was further spun at 12,000 rpm for 10 min at 4 °C; the pellets obtained from this step were sonicated in protein lysis buffer (B) containing 50 mM Tris HCl (pH 7.5) (Sigma Aldrich, St Louis, MO, USA), 150 mM NaCl (POCH Basic, Gliwice, Poland), 1% Triton X-100 (Bioshop, Burlington, OA, Canada), 0.1% SDS ((Sigma Aldrich, St. Louis, MO, USA), 2 mM EDTA, 1 mM NaVO3, 5 mM NaF and 1X protease inhibitor cocktail. Protein concentrations were measured using a BCA kit (Thermo Scientific, Rockford, IL, USA)). Next, the proteins were separated by SDS-PAGE and transferred to nitrocellulose membranes and blocked for 1 h in 1% blocking solution [BM Chemiluminescence Western Blotting Kit (Mouse/Rabbit);Mannheim Germany]. After blocking, the membranes were incubated overnight at 4 °C with the respective primary antibodies. The following antibodies were used: total and phospho-ERK (both at a dilution of 1:1000, phospho-Millipore, Temecula, CA, USA, total-Cell Signaling, Leiden, Netherlands), total and phospho-p70S6K (pp70S6K, Thr389) (respectively 1:1000 and 1:500, Abcam, Cambridge, UK), synapsin I (Abcam, Cambridge, UK 1:1000), GluA1 AMPA (Abcam, Cambridge, UK, 1:1000), β-actin (Sigma Aldrich, St. Louis, MO, USA, 1:12000). For the determination of the levels of total ERK and β-actin we used restore plus stripping buffer (Thermo Scientific, Rockford, IL, USA). The following day, the membranes were washed three times for 10 min in Tris-buffered saline with Tween (TBS-T) and incubated for 30 min with anti-mouse/anti-rabbit-IgG-peroxidase conjugated antibodies. This set of secondary antibodies was also a component of the BM Chemiluminescence Western Blotting Kit (Mouse/Rabbit) (Roche, Mannheim, Germany). After incubation, the membranes were washed three times for 10 min with TBS-T. In the last step, the blots were incubated with a detection reagent (Roche, Mannheim, Germany). The signals from the tested proteins were visualized and measured using a Fuji-Las1000 system and Fuji Image Gauge v.4.0 software. To check for transfer and loading, β-actin was indicated on each blot. Each signal from the phospho and total protein was divided by its corresponding β-actin signal to arrive at the final result (ratio of the optical density of a particular protein to that of β-actin). The data on the graph are expressed as % of change vs. control.

### 4.8. Morphological Analysis of Dendritic Spines

In order to quantify dendritic spines, Golgi staining was performed using the FD Rapid GolgiStainTM Kit (FD Neuro Technologies, Inc., Columbia, MD, USA) according to the manufacturer’s instructions. Three hours after i.p. administration of Ro, treated and vehicle rats were deeply anesthetized with pentobarbital, their brains were removed from the skull and rinsed in double-distilled water. Brains were immersed in impregnation solution, made by mixing equal volumes of Solutions A and B, overnight and then stored in fresh solution at room temperature (RT) in the dark for 2 weeks. Next, brains were transferred into Solution C overnight and then stored in fresh solution at RT for 1 week in the dark. The brains were frozen on dry ice and sliced into 100 μm-thick frontal sections using a freezing microtome (Reichert (N° 17169), Wien, Austria). The sections were mounted on gelatin-coated slides (FD Neuro Technologies, Inc., Columbia, MD, USA). Dried sections were processed according to the manufacturer’s instructions using Solutions D and E provided in the kit. The dendritic spines were counted on the secondary and tertiary apical dendrites of neurons in the Cg3 and IL of the medial prefrontal cortex (mPFC) using a light microscope (Leica, DMLB; Leica, Denmark) equipped with a projecting camera (Basler Vision Technologies, Germany) and a microscope stage connected to an xyz stepper (PRIOR ProScan) controlled by a computer using Visiopharm New CAST software (Visiopharm, Denmark). The analyzed area was outlined under low magnification (×5), while spine counting was performed under ×100 magnification within three stained sections from each animal (AP = 3.20 to 2.20 mm from bregma) [56] using a randomized meander sampling. To ascertain relative spine density, spines on multiple dendritic branches were counted to obtain an average spine number per 10 µm. The average value for each region, in each rat, was obtained, and five to six rats were analyzed per group. Microphotographs were taken using a light microscope Nikon Eclipse E600 (Nikon, Japan), equipped with a black and white camera (Leica Microsystems CMS, GmbH Germany) connected to a computer running the Leica Application Suite (LAS) version 4.5 software.

### 4.9. Statistical Analysis

Data are presented as means ± SEM and evaluated using two way-ANOVA and Newman–Keuls multiple comparison post hoc tests when appropriate. *p* < 0.05 was considered significant.

## Figures and Tables

**Table 1:** Effect of zinc deficient diet (ZnD) and Ro 25–6981 and ketamine treatment on the locomotor activity of rats.

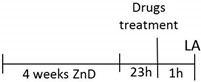	**Experimental Group**	**Locomotor Activity (10 min)**
	ZnA + NaCl	100 ± 9.28
	ZnA + Ro 25–6981 ZnA + Ketamine	137.8 ± 39.01 76.22 ± 14.6
	ZnD + NaCl	70.76 ± 5.03
	ZnD + Ro 25–6981 ZnD + Ketamine	124.56 ± 12.3 66.93 ± 4.87

## References

[1] Busfield J. (2012). Challenging claims that mental illness has been increasing and mental well-being declining. Soc. Sci. Med..

[2] Hidaka B.H. (2012). Depression as a disease of modernity: Explanations for increasing prevalence. J. Affect. Disord..

[3] Wittchen H.U., Jacobi F., Rehm J., Gustavsson A., Svensson M., Jonsson B., Olesen J., Allgulander C., Alonso J., Faravelli C. (2011). The size and burden of mental disorders and other disorders of the brain in Europe 2010. Eur. Neuropsychopharmacol..

[4] Rosenblat J.D., McIntyre R.S., Alves G.S., Fountoulakis K.N., Carvalho A.F. (2015). Beyond Monoamines-Novel Targets for Treatment-Resistant Depression: A Comprehensive Review. Curr. Neuropharmacol..

[5] Szewczyk B., Szopa A., Serefko A., Poleszak E., Nowak G. (2018). The role of magnesium and zinc in depression: Similarities and differences. Magnes. Res..

[6] Vashum K.P., McEvoy M., Milton A.H., McElduff P., Hure A., Byles J., Attia J. (2014). Dietary zinc is associated with a lower incidence of depression: Findings from two Australian cohorts. J. Affect. Disord..

[7] Jacka F.N., Maes M., Pasco J.A., Williams L.J., Berk M. (2012). Nutrient intakes and the common mental disorders in women. J. Affect. Disord..

[8] Hansen C.R., Malecha M., Mackenzie T.B., Kroll J. (1983). Copper and zinc deficiencies in association with depression and neurological findings. Biol. Psychiatry.

[9] Maes M., Bosmans E., De Jongh R., Kenis G., Vandoolaeghe E., Neels H. (1997). Increased serum IL-6 and IL-1 receptor antagonist concentrations in major depression and treatment resistant depression. Cytokine.

[10] Lehto S.M., Ruusunen A., Tolmunen T., Voutilainen S., Tuomainen T.P., Kauhanen J. (2013). Dietary zinc intake and the risk of depression in middle-aged men: A 20-year prospective follow-up study. J. Affect. Disord..

[11] Marcellini F., Giuli C., Papa R., Gagliardi C., Dedoussis G., Herbein G., Fulop T., Monti D., Rink L., Jajte J. (2006). Zinc status, psychological and nutritional assessment in old people recruited in five European countries: Zincage study. Biogerontology.

[12] Markiewicz-Zukowska R., Gutowska A., Borawska M.H. (2015). Serum zinc concentrations correlate with mental and physical status of nursing home residents. PLoS ONE.

[13] Siwek M., Szewczyk B., Dudek D., Styczen K., Sowa-Kucma M., Mlyniec K., Siwek A., Witkowski L., Pochwat B., Nowak G. (2013). Zinc as a marker of affective disorders. Pharmacol. Rep..

[14] McLoughlin I.J., Hodge J.S. (1990). Zinc in depressive disorder. Acta Psychiatr. Scand..

[15] Maes M., De Vos N., Demedts P., Wauters A., Neels H. (1999). Lower serum zinc in major depression in relation to changes in serum acute phase proteins. J. Affect. Disord..

[16] Siwek M., Dudek D., Schlegel-Zawadzka M., Morawska A., Piekoszewski W., Opoka W., Zieba A., Pilc A., Popik P., Nowak G. (2010). Serum zinc level in depressed patients during zinc supplementation of imipramine treatment. J. Affect. Disord..

[17] Mlyniec K., Davies C.L., Budziszewska B., Opoka W., Reczynski W., Sowa-Kucma M., Doboszewska U., Pilc A., Nowak G. (2012). Time course of zinc deprivation-induced alterations of mice behavior in the forced swim test. Pharmacol. Rep..

[18] Mlyniec K., Nowak G. (2012). Zinc deficiency induces behavioral alterations in the tail suspension test in mice. Effect of antidepressants. Pharmacol. Rep..

[19] Whittle N., Lubec G., Singewald N. (2009). Zinc deficiency induces enhanced depression-like behaviour and altered limbic activation reversed by antidepressant treatment in mice. Amino Acids.

[20] Doboszewska U., Sowa-Kucma M., Mlyniec K., Pochwat B., Holuj M., Ostachowicz B., Pilc A., Nowak G., Szewczyk B. (2015). Zinc deficiency in rats is associated with up-regulation of hippocampal NMDA receptor. Prog. Neuropsychopharmacol. Biol. Psychiatry.

[21] Doboszewska U., Szewczyk B., Sowa-Kucma M., Mlyniec K., Rafalo A., Ostachowicz B., Lankosz M., Nowak G. (2015). Antidepressant activity of fluoxetine in the zinc deficiency model in rats involves the NMDA receptor complex. Behav. Brain Res..

[22] Tassabehji N.M., Corniola R.S., Alshingiti A., Levenson C.W. (2008). Zinc deficiency induces depression-like symptoms in adult rats. Physiol. Behav..

[23] Yang C., Yang J., Luo A., Hashimoto K. (2019). Molecular and cellular mechanisms underlying the antidepressant effects of ketamine enantiomers and its metabolites. Transl. Psychiatry.

[24] Zarate C.A., Niciu M.J. (2015). Ketamine for depression: Evidence, challenges and promise. World Psychiatry.

[25] Berman R.M., Cappiello A., Anand A., Oren D.A., Heninger G.R., Charney D.S., Krystal J.H. (2000). Antidepressant effects of ketamine in depressed patients. Biol. Psychiatry.

[26] Zarate C.A., Singh J.B., Carlson P.J., Brutsche N.E., Ameli R., Luckenbaugh D.A., Charney D.S., Manji H.K. (2006). A randomized trial of an N-methyl-D-aspartate antagonist in treatment-resistant major depression. Arch. Gen. Psychiatry.

[27] Daly E.J., Singh J.B., Fedgchin M., Cooper K., Lim P., Shelton R.C., Thase M.E., Winokur A., Van Nueten L., Manji H. (2018). Efficacy and Safety of Intranasal Esketamine Adjunctive to Oral Antidepressant Therapy in Treatment-Resistant Depression: A Randomized Clinical Trial. JAMA Psychiatry.

[28] DiazGranados N., Ibrahim L.A., Brutsche N.E., Ameli R., Henter I.D., Luckenbaugh D.A., Machado-Vieira R., Zarate C.A. (2010). Rapid resolution of suicidal ideation after a single infusion of an N-methyl-D-aspartate antagonist in patients with treatment-resistant major depressive disorder. J. Clin. Psychiatry.

[29] Canuso C.M., Singh J.B., Fedgchin M., Alphs L., Lane R., Lim P., Pinter C., Hough D., Sanacora G., Manji H. (2018). Efficacy and Safety of Intranasal Esketamine for the Rapid Reduction of Symptoms of Depression and Suicidality in Patients at Imminent Risk for Suicide: Results of a Double-Blind, Randomized, Placebo-Controlled Study. Am. J. Psychiatry.

[30] De Berardis D., Fornaro M., Valchera A., Cavuto M., Perna G., Di Nicola M., Serafini G., Carano A., Pompili M., Vellante F. (2018). Eradicating Suicide at Its Roots: Preclinical Bases and Clinical Evidence of the Efficacy of Ketamine in the Treatment of Suicidal Behaviors. Int. J. Mol. Sci..

[31] De Berardis D., Tomasetti C., Pompili M., Serafini G., Vellante F., Fornaro M., Valchera A., Perna G., Volpe U., Martinotti G. (2020). An Update on Glutamatergic System in Suicidal Depression and on the Role of Esketamine. Curr. Top. Med. Chem..

[32] Li N., Liu R.J., Dwyer J.M., Banasr M., Lee B., Son H., Li X.Y., Aghajanian G., Duman R.S. (2011). Glutamate N-methyl-D-aspartate receptor antagonists rapidly reverse behavioral and synaptic deficits caused by chronic stress exposure. Biol. Psychiatry.

[33] Yang B.K., Qin J., Nie Y., Chen J.C. (2018). Sustained antidepressant action of the N-methyl-D-aspartate receptor antagonist MK-801 in a chronic unpredictable mild stress model. Exp. Ther. Med..

[34] Cunningham M.G., Ames H.M., Christensen M.K., Sorensen J.C. (2007). Zincergic innervation of medial prefrontal cortex by basolateral projection neurons. Neuroreport.

[35] Frederickson C.J., Danscher G. (1990). Zinc-containing neurons in hippocampus and related CNS structures. Prog. Brain Res..

[36] Maret W. (2013). Zinc biochemistry: From a single zinc enzyme to a key element of life. Adv. Nutr..

[37] Suh S.W., Chen J.W., Motamedi M., Bell B., Listiak K., Pons N.F., Danscher G., Frederickson C.J. (2000). Evidence that synaptically-released zinc contributes to neuronal injury after traumatic brain injury. Brain Res..

[38] Mlyniec K., Gawel M., Nowak G. (2015). Study of antidepressant drugs in GPR39 (zinc receptor(-)/(-)) knockout mice, showing no effect of conventional antidepressants, but effectiveness of NMDA antagonists. Behav. Brain Res..

[39] Li N., Lee B., Liu R.J., Banasr M., Dwyer J.M., Iwata M., Li X.Y., Aghajanian G., Duman R.S. (2010). mTOR-dependent synapse formation underlies the rapid antidepressant effects of NMDA antagonists. Science.

[40] Pochwat B., Rafalo-Ulinska A., Domin H., Misztak P., Nowak G., Szewczyk B. (2017). Involvement of extracellular signal-regulated kinase (ERK) in the short and long-lasting antidepressant-like activity of NMDA receptor antagonists (zinc and Ro 25-6981) in the forced swim test in rats. Neuropharmacology.

[41] Doboszewska U., Szewczyk B., Sowa-Kucma M., Noworyta-Sokolowska K., Misztak P., Golebiowska J., Mlyniec K., Ostachowicz B., Krosniak M., Wojtanowska-Krosniak A. (2016). Alterations of Bio-elements, Oxidative, and Inflammatory Status in the Zinc Deficiency Model in Rats. Neurotox. Res..

[42] Paoletti P., Vergnano A.M., Barbour B., Casado M. (2009). Zinc at glutamatergic synapses. Neuroscience.

[43] Pochwat B., Nowak G., Szewczyk B. (2019). An update on NMDA antagonists in depression. Expert Rev. Neurother.

[44] Ghosal S., Duman C.H., Liu R.J., Wu M., Terwilliger R., Girgenti M.J., Wohleb E., Fogaca M.V., Teichman E.M., Hare B. (2019). Ketamine rapidly reverses stress-induced impairments in GABAergic transmission in the prefrontal cortex in male rodents. Neurobiol. Dis..

[45] Talbot J.N., Geffert L.M., Jorvig J.E., Goldstein R.I., Nielsen C.L., Wolters N.E., Amos M.E., Munro C.A., Dallman E., Mereu M. (2016). Rapid and sustained antidepressant properties of an NMDA antagonist/monoamine reuptake inhibitor identified via transporter-based virtual screening. Pharmacol. Biochem. Behav..

[46] Frazzini V., Granzotto A., Bomba M., Massetti N., Castelli V., d’Aurora M., Punzi M., Iorio M., Mosca A., Delli Pizzi S. (2018). The pharmacological perturbation of brain zinc impairs BDNF-related signaling and the cognitive performances of young mice. Sci. Rep..

[47] Christoffel D.J., Golden S.A., Russo S.J. (2011). Structural and synaptic plasticity in stress-related disorders. Rev. Neurosci..

[48] Krzystyniak A., Baczynska E., Magnowska M., Antoniuk S., Roszkowska M., Zareba-Koziol M., Das N., Basu S., Pikula M., Wlodarczyk J. (2019). Prophylactic Ketamine Treatment Promotes Resilience to Chronic Stress and Accelerates Recovery: Correlation with Changes in Synaptic Plasticity in the CA3 Subregion of the Hippocampus. Int. J. Mol. Sci..

[49] Duman C.H., Duman R.S. (2015). Spine synapse remodeling in the pathophysiology and treatment of depression. Neurosci. Lett..

[50] Goldin M., Segal M. (2003). Protein kinase C and ERK involvement in dendritic spine plasticity in cultured rodent hippocampal neurons. Eur. J. Neurosci..

[51] Ota K.T., Liu R.J., Voleti B., Maldonado-Aviles J.G., Duric V., Iwata M., Dutheil S., Duman C., Boikess S., Lewis D.A. (2014). REDD1 is essential for stress-induced synaptic loss and depressive behavior. Nat. Med..

[52] Kumar V., Zhang M.X., Swank M.W., Kunz J., Wu G.Y. (2005). Regulation of dendritic morphogenesis by Ras-PI3K-Akt-mTOR and Ras-MAPK signaling pathways. J. Neurosci..

[53] Yang C., Ren Q., Qu Y., Zhang J.C., Ma M., Dong C., Hashimoto K. (2018). Mechanistic Target of Rapamycin-Independent Antidepressant Effects of (R)-Ketamine in a Social Defeat Stress Model. Biol. Psychiatry.

[54] Yang C., Shirayama Y., Zhang J.C., Ren Q., Yao W., Ma M., Dong C., Hashimoto K. (2015). R-ketamine: A rapid-onset and sustained antidepressant without psychotomimetic side effects. Transl. Psychiatry.

[55] Szewczyk B., Pochwat B., Rafalo A., Palucha-Poniewiera A., Domin H., Nowak G. (2015). Activation of mTOR dependent signaling pathway is a necessary mechanism of antidepressant-like activity of zinc. Neuropharmacology.

[56] Paxinos G., Watson C. (1986). The Rat Brain in Stereotaxic Coordinates.

